# Intergenerational Mealtimes in Adult Day Care Settings: Impact of a Pilot Randomised Control Study on the Well-Being, Health, and Food Intake of Older Adults

**DOI:** 10.3390/healthcare14050635

**Published:** 2026-03-03

**Authors:** Raúl López-López, Reyes Artacho, Celia Rodríguez-Pérez, Judith Justicia-García, Alicia Carrillo, Mariano Sánchez

**Affiliations:** 1 Department of Sociology, University of Granada, 18071 Granada, Granada, Spain; marianos@ugr.es; 2 Department of Nutrition and Food Science, Faculty of Pharmacy, University of Granada, 18071 Granada, Granada, Spain; rartacho@ugr.es (R.A.); celiarp@ugr.es (C.R.-P.); 3 Institute of Nutrition and Food Technology (INYTA) ‘José Mataix’, Biomedical Research Centre, University of Granada, Avda. del Conocimiento s/n, 18100 Granada, Granada, Spain; 4 Instituto de Investigación Biosanitaria ibs. GRANADA, Avenida de Madrid, 15, 18012 Granada, Granada, Spain; 5 Nestlé España S.A., 08950 Esplugues de Llobregat, Barcelona, Spain; judith.justicia@es.nestle.com; 6 Macrosad, Parque Científico y Tecnológico Geolit, Av. De la Innovación, 3, 23260 Mengíbar, Jaén, Spain; direccion@macrosad.com; 7 Macrosad Chair in Intergenerational Studies, Faculty of Political Sciences and Sociology, University of Granada, 18071 Granada, Granada, Spain

**Keywords:** intergenerational commensality, older adults, subjective well-being, self-esteem, perceived health, leftover food, mixed methods, pilot randomised controlled trial

## Abstract

**Background/Objectives**: Empirical evidence on intergenerational commensality in institutional care settings remains scarce. This pilot and feasibility study evaluated the preliminary impact of an innovative intergenerational mealtime model on older adults’ subjective well-being, self-esteem, perceived health, and food consumption in an adult day care setting. **Methods**: A 16-week wait-list randomised controlled pilot trial with a sequential explanatory mixed-methods design was conducted in an intergenerational centre in southern Spain. Twenty-two older adults who reside in a community living setting and attend a day care service were randomly assigned to two intergenerational dining intervention/waiting-list groups or a control group. Participants in the intervention/waiting-list groups had lunch four times per week with children 2–3 years of age, following a structured protocol. The quantitative outcomes examined included subjective well-being (WHO-5 Well-Being Index), self-esteem (Rosenberg scale), perceived health (EuroQol EQ-5D), and objective assessment of plate leftovers using photographic records and the Comstock visual estimation method. Qualitative data were collected through semi-structured interviews and ethnographic observation. The trial was registered at ClinicalTrials.gov (NCT06996418). **Results**: Across the study period, intervention, waiting-list, and control groups showed preliminary improvements in subjective well-being and self-esteem, but with no significant group-by-time interaction. In contrast, mixed-effects models revealed an encouraging significant reduction in plate leftovers among older adults during intergenerational meals, particularly in second courses. The reduction was consolidated during the post-intervention follow-up. Qualitative findings showed perceived improvements in emotional well-being, motivation, and appetite, thus highlighting potential relational and affective mechanisms underlying changes in eating behaviour. **Conclusions**: This pilot study shows promise for intergenerational commensality in adult day care settings and provides preliminary evidence of its potential to promote well-being and self-esteem and reduce food waste among older adults. Larger, multi-centre, appropriately powered trials are warranted to validate these findings.

## 1. Introduction

Population ageing is one of the most significant demographic phenomena of the 21st century. It is posing substantial challenges to health systems, social services, and long-term care models [1,2]. Naturally, Spain is not exempt from this ageing trend [3]. One result of this demographic shift often goes unnoticed. As longevity increases—and fertility is at least partially maintained—different age cohorts will be contemporary for longer periods of time. Longer-living societies are therefore also more multigenerational [4], particularly in contexts where kinship networks are becoming more vertically extended.

Consequently, and especially considering the principle of linked lives, a core tenet of the life course perspective [5], it makes sense to study the health, well-being, and long-term care of older adults in relation to their connections with younger generations. At the family level, it is easy to understand that a family unit with only two generations will develop its long-term care plan with more limitations than a more multigenerational family. This example can be transferred to the societal level, moving from the intra-family to the extra-family sphere, since not all living kin are necessarily available kin [6]. This underlines the importance of cultivating extra-familial intergenerational connections, as they promote bonds that can form the basis of mutual support and care relationships. On this ground, this paper presents outputs from a new, promising model for fostering such connections: an intergenerational dining room located in a day centre for older adults.

Day centres provide comprehensive daytime care to older adults with the aim of maintaining and improving personal autonomy and functional independence. In Spain, it is rare for a day centre to share facilities with a nursery school as part of an intergenerational centre, but the few examples of this type of co-location that do exist make it possible to study innovative ways of promoting the health and well-being of older adults. Intergenerational centres are designed to intentionally facilitate intergenerational relationships while offering services for the development and well-being of people belonging to different generations [7].

### Intergenerational Commensality

Commensality refers to the table companions who, by eating together, create social bonds and establish community [8]. If we add the adjective intergenerational and place ourselves in a day centre for older people, it may be worth exploring whether the practice of intergenerational commensality could be a useful innovation for improving the quality of such care. Despite the paucity of research on intergenerational commensality [9], it has been suggested that this type of shared meal experience can counter ageism and the institutionalisation of age segregation [9].

Intergenerational programmes focusing on nutrition—an area which might be considered close to intergenerational commensality—are a novel approach [10,11]. These programmes have their own set of practices and show quite promising development, compared to other nutrition improvement programmes aimed separately at children and older adults [12,13]. In fact, intergenerational programmes have been proven to improve nutritional knowledge and behaviours among participants [10,11].

However, while previous studies have examined intergenerational nutrition education programmes, rigorous experimental trials assessing the impact of daily intergenerational commensality in institutional care settings remain virtually absent. This lack of research makes it difficult to develop new evidence-based practices, policies and programmes. Hence, our decision to undertake a pilot randomised control trial: we saw an opportunity to create a new model of an intergenerational dining daily experience. The model was created using design thinking, but the process is not described herein as it is beyond the scope of this article. Our intention was to design and then conduct an initial study to understand, on a small scale, the feasibility and possible impacts of the model, as a preliminary step to deciding whether to conduct a full randomised study.

Three entities (the University of Granada, the Macrosad cooperative and Nestlé España S.A.) worked together to co-create a model named the ‘Intergenerational Dining Room’ (IGD). The IGD model is based on the following four pillars: (1) relational care, which conceives well-being as the product of bonds of support, reciprocity, and mutual recognition [14,15]; (2) eating together/commensality, a social practice that places food at the heart of its emotional and communal dimensions (conviviality and communality, respectively) [16]; (3) the life course, which views the intertwining (relationships) between generations as an opportunity to link moments, trajectories, and transitions throughout life [17]; and (4) experiential nutrition and food education, by which eating together becomes a time for situated learning [9,12].

Based on the foregoing, one objective of the pilot study was to assess the effect of the IGD model on the perceived health, subjective well-being, self-esteem, and food intake of older adults in a day care setting. These specific effects were selected because they represent age-group dimensions regarding how old age is experienced by participating older adults [9].

## 2. Materials and Methods

### 2.1. Study Design

The pilot and feasibility study was conducted as a wait-list randomised controlled trial with three arms: intervention group, waiting-list group and control group. The design was approved by the Ethics Committee of the University of Granada (registration number: 4592/CEIH/2024) and its protocol was registered retrospectively at ClinicalTrials.gov (Identifier: NCT06996418), where the trial protocol and statistical analysis plan can be viewed. Retrospective registration—participant enrolment started in January 2025 whereas registration took place in May 2025—was due to internal timing issues to do with information processing from the IGD model. No relevant implications of retrospective registration affecting the study design and outcomes were identified. In addition, the study design, development, and reporting process followed the guidelines indicated by CONSORT for reporting pilot and feasibility trials [18].

The pilot consisted of launching a planned IGD designed from scratch as a structured space and time for intergenerational dining to be shared by older adults and children attending, respectively, the day care centre and the nursery school at the Macrosad Intergenerational Reference Centre in Albolote (Spain). The study employed a mixed sequential-explanatory methodology [19], combining longitudinal quantitative analyses with qualitative strategies such as ethnographic observation, interviews, and thematic analysis [20,21].

Assessment timepoints were operationalized as follows: baseline measurements were conducted in the beginning of IGD exposure (week 1); the intermediate assessment corresponded to the completion of the intervention group’s period in the IGD (week 9); the endline assessment was conducted after the IGD stay by the waiting-list group (week 17); and follow-up measurements of leftover food were obtained in the 2 weeks after the intervention and waiting-list groups had left the IGD (weeks 9–10 and 17–18). This structure reflects the staggered implementation inherent to the wait-list design.

### 2.2. Intervention

The intervention lasted a total of 16 weeks—plus two follow-up weeks—that were divided into two 8-week blocks due to the IGD’s limited space. In each of the two blocks, 5 older adults from the day centre—referred to as ‘Older Friends’ (OFs) in the context of the intergenerational centre—out of a total of 22 (mean age $\bar{x}$ = 82.8 ± 4.6) selected at random, ate lunch four times a week with 5 children—‘Little Friends’ (LFs)—from a total of 22 (mean age $\bar{x}$ = 2.1 ± 0.3) belonging to two random classrooms in the nursery school. Participating children were selected randomly as well. The reasons for including children aged 2–3 years were twofold. On the one hand, the age span in the nursery was limited to 0–3 years. On the other hand, and according to nursery teachers, 2–3-year-old children demonstrated spontaneous affective interaction and observational learning skills that might enhance reciprocal engagement during meals. According to the Ethics Committee guidelines, both OFs and parents/legal guardians of LFs provided written informed consent prior to the inclusion of older adults and children in the study.

The intervention consisted of a total of 64 intergenerational lunches lasting an average of 40 min, plus 24 sessions of fully peer-based dining. The intergenerational lunches were divided into three sequential stages, as follows: (1) before the meal: welcome and greeting ritual, joint hand hygiene, and micro-activity on food education using Nutriplato^®^—e.g., identification of foods, food textures or colours, etc. [22]; (2) during the meal: OFs–LFs were seated in an intercalated manner, rotating tasks—setting the table, teaching, helping—were performed in OF–LF pairs, and eating and social interactions were facilitated on-site by two intergenerational practitioners; (3) after the meal: joint tidying up, recycling, and closing ritual. The older adults and children in the control group had their lunch in their regular peer-based dining rooms. Instructions were given so that every day the same menu was served in both the IGD and the peer-based dining rooms.

Given the pilot and feasibility nature of the study, no formal a priori sample size calculation was conducted. The number of participants was determined by the operational capacity of the intergenerational centre and the need to assess feasibility parameters (e.g., recruitment, retention, and intervention implementation).

### 2.3. Instruments

Demographic data collected for the study included: age, sex, and length of time attending day care centre (months).

Anthropometric measurements and nutritional status included weight (kg), height (cm), and calf circumference (CC, cm). These measurements were conducted in the morning, with participants wearing light clothing and no footwear. All these measurements followed the standardized procedures of the International Society for the Advance of Kinanthropometry (ISAK) to ensure accuracy, reliability, and comparability [23]. Body mass index (BMI) was calculated from weight and height (kg/m^2^). Based on BMI, OFs were classified as underweight (<22 kg/m^2^), normal weight (22–26.9 kg/m^2^), overweight (27–29.9 kg/m^2^), or obese (≥30 kg/m^2^) [24]. A CC value < 31 cm was associated with an increased risk of malnutrition in older adults [25].

Nutritional status was assessed using the Mini Nutritional Assessment-Short Form (MNA-SF) [26]. The MNA-SF consists of six items related to appetite, weight loss, mobility, acute illness, neuropsychological problems, and BMI or CC. Total scores ranged from 0 to 14 points; scores of 12–14 indicated normal nutritional status, scores of 8–11 indicated risk of malnutrition, and scores below 8 indicated malnutrition. The Eating Assessment Tool-10 (EAT-10) was also employed to screen for the possible presence of dysphagia [27].

An ad hoc questionnaire was used to collect quantitative and qualitative information on well-being, self-esteem, and health among OFs. To obtain further quantitative data, three instruments were used: the WHO-5 Well-Being Index (WHO-5) [28] to assess subjective well-being, the Rosenberg Scale [29] for self-esteem, and the EuroQol EQ-5D (EQ-5D) visual analogue scale [30] to assess self-perceived health. This questionnaire was administered on three occasions, during (weeks 1–3 and 9) and after the experiment (week 17).

Each OF was interviewed following a semi-structured script (available as Supplementary Materials) that included a section on physical and emotional well-being with questions such as: How do you feel before, during, and after meals with the LFs? Is it the same as when you are eating with your peers at the day centre? Would you say that this experience has influenced your mood or general health? These interviews were conducted during weeks 4–5 and 9 in the first block and during weeks 13 and 17 in the second block.

Regarding food consumption, OFs’ food leftovers were measured both at the IGD and in the two dining rooms at the day centre (DCD). Specifically, photographs were taken of the first and second courses of 56 meals at the IGD—it was not possible to cover 100% of the sessions due to various errors in the information collection process (incorrect plate labelling, plates being washed prematurely) or unexpected circumstances (technical problems, staff availability issues)—and 77 meals in the DCD. All images were taken with an iPhone XR (Apple Inc., Cupertino, CA, USA) positioned at a 90° angle directly above the plate, mounted on a tripod at a fixed height of 75 cm. All images were captured on a uniform brown table surface and using standardised white plates. Lighting conditions were kept stable and consistent: all photographs were taken in the same dining area under indoor ambient light, complemented when necessary by a 10.2” high tripod ring light (LIPETY, Quanzhou, China) offering continuous illumination with adjustable brightness and colour temperature (3200–6500 K), free of flicker, glare, or ghosting. Plates were photographed immediately after the meal, and no leftovers were altered before image acquisition.

The dishes were classified according to their main ingredient following the Nutriplato^®^ guide: first courses (rice, pasta, potatoes, or legumes) and second courses (meat, fish, or eggs with vegetables). Each photograph was taken by the same evaluator, and the data were analysed independently by three assessors with prior training in Comstock visual estimation scale [31]. Assessors blinding was only partial: the identity of the assessed participants was blinded, but not the levels of remaining food assigned by each assessor. For each dish served (first and second courses), leftover food was assessed through the proportion of food remaining on the plate, which was categorized as one of five levels: 0% (no waste), 25%, 50%, 75%, and 100% (full waste). The reliability of the photographic method was measured using the intraclass correlation coefficient, which determined that reliability was very high—ICC(2,k) = 0.98. Disagreements undermining reliability were resolved through discussion with a fourth investigator.

### 2.4. Participants

A total of 22 older adults were selected to participate in the study and were randomly assigned to either the intervention/waiting-list or the control groups. For descriptive purposes, intervention and waiting-list groups were combined, as both corresponded to participants exposed to the IGD during different phases of the wait-list design. The participant allocation ratio was 1:1. Random allocation and sequence were computer generated and implemented by an independent researcher not involved in the day care centre. Randomization was not stratified by sex due to the small sample size inherent to the pilot design, which might have contributed to the observed sex imbalance between groups. On average, older participants were aged 82.8 years old, went regularly to the day care centre (attendance ≥ 3 days/week), and made use of the daily lunch service. Other inclusion criteria were having sufficient functional capacity to eat independently or with minimal support (e.g., ability to sit stably, use basic utensils, and participate in meals without continuous assistance) as per assessment by day care staff, having sufficient cognitive ability for basic social interaction (GDS ≤ 5), and signed informed consent—obtained after acceptance in study, once centre staff had invited all older adults meeting inclusion criteria to participate if they wished. Older people with severe behavioural disorders (e.g., intense agitation, aggression, or persistent disruptive behaviours) that might compromise the safety or relational climate of the group were not included. In addition, all participants were checked for eating-related risk of dysphagia.

## 3. Data Analysis

The data collected using the WHO-5, Rosenberg Scale, and EQ-5D instruments were analysed using mixed linear models, which are suitable for treating repeated measures with a longitudinal structure and unbalanced data. A descriptive exploration of the scores had been carried out previously, using means and standard deviations, stratified by group (intervention, waiting-list, and control-groups) and by exposure phase (baseline–endline) (Table 1). Given the wait-list design of the intervention, time was operationalised as time relative to exposure, allowing for a comparison of pre-post changes between OFs exposed and not exposed to IGD in each period.

The models included random intercepts at the individual level, and in the case of OFs, they were adjusted for age and cognitive impairment—using the Reisberg Global Deterioration Scale (GDS)—as covariates. For each dependent variable, fixed effects of time, group, and their interaction were estimated, as well as post hoc contrasts and estimated marginal means.

The data obtained from the images of the plate leftovers and the application of the Comstock visual scale were analysed using REML-adjusted mixed linear models, with a random intercept for each participant. These models grouped the data from 44 OFs and LFs to enhance statistical stability and estimation precision. Preliminary analyses restricted to a single cohort (e.g., OF, *n* = 22) resulted in reduced model stability and less reliable variance estimates. By modelling both cohorts jointly and including cohort and interaction terms, improved parameter estimation was warranted while retaining the ability to derive cohort-specific effects through estimated marginal means.

Before estimating the different models, a systematic analysis of the missing data in the variables of interest was carried out, given the not insignificant volume of incomplete observations inherent in fieldwork. Approximately 28% of observations for both first- and second-course plate waste were missing. Little’s Missing Completely At Random (MCAR) test was non-significant—X^2^(2) = 1.21, *p* = 0.547—indicating that the missing data pattern was compatible with a MCAR mechanism. Given that the longitudinal mixed-effects modeling framework used in the analyses were estimated via restricted maximum likelihood, the models provide unbiased parameter estimates under MCAR assumptions. Therefore, no multiple imputation procedure was applied. Although complete-case analysis may reduce statistical power and slightly increase standard errors, this approach was considered appropriate given the pilot and feasibility nature of the study.

For each outcome (first course leftover food, second course leftover food, and mean plate leftovers), the effects on participants exposed to the IGD (intervention and waiting-list groups) versus control participants as well as its possible variation over time were modelled using a continuous time term τ rescaled and centred that was incorporated linearly and quadratically. The models were adjusted by controlling for gender and, specifically in OFs, for age, cognitive impairment (GDS), and nutritional status (MNA-SF) as covariates. The significance of fixed effects was assessed using type III ANOVA and the Kenward–Roger correction. To communicate the effects, estimated marginal means (EMMs), and contrasts between groups at key points in the period (e.g., start/end) were obtained. Finally, for post-intervention follow-up, this strategy was replicated by replacing the phase and refocusing the time in order to assess whether the pattern of reduction in OF’s mean plate leftovers was stable or if it progressed after leaving the IGD. All quantitative analyses were conducted using R version 4.5.2 (R Foundation for Statistical Computing, Vienna, Austria).

Regarding qualitative data, Knoblauch’s focused ethnography standards were applied [21]. Qualitative materials were transcribed and analysed using thematic analysis [20], with NVivo 14 (QSR International, Melbourne, Australia) as a supporting tool. The analytical process was iterative and included initial inductive coding and the progressive development of themes, integrating data from interviews, observations and field notes in order to enrich the interpretation and contextualise the meanings that emerged.

## 4. Results

### 4.1. Participant Flow and Adherence

**Figure 1:**
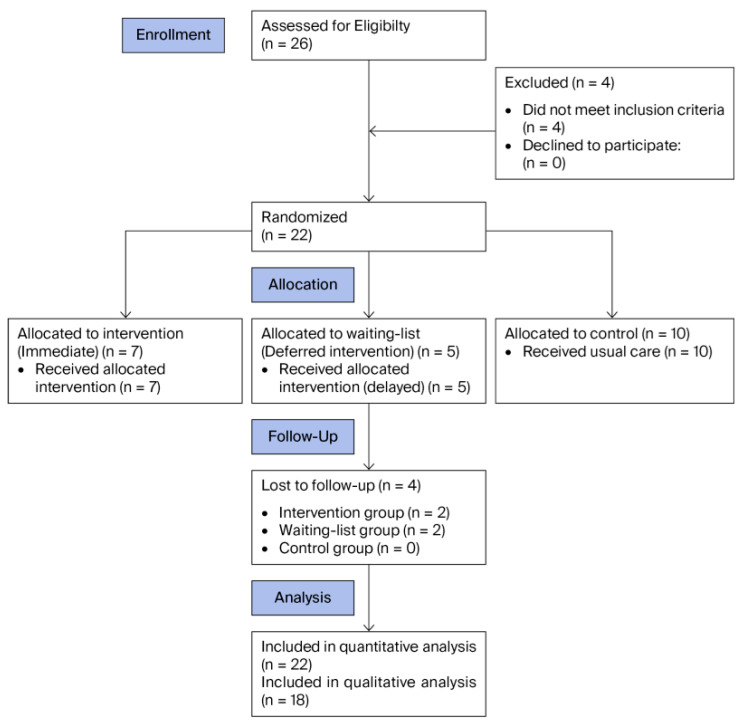
Flow diagram.

Figure 1 shows the flow of participants through the study, including eligibility, randomization, follow-up, and final analysis. Eighty-two percent of OFs completed the intervention and follow-up assessments. Adherence to the intervention was quantified by the number of sessions attended. On average, OFs attended 80% of all 88 pilot sessions. No adverse events, other than withdrawals, were reported during the study period. Recruitment took place between January and February 2025. The final follow-up period was from 24 June to 11 July 2025.

### 4.2. Sample Characteristics

Sociodemographic, nutritional, and cognitive and anthropometric characteristics of older participants are presented in Table 1.

The study population consisted of 22 older adults with a mean age of 82.8 ± 4.6 years. Women were in the majority, accounting for 58.3% of the intervention and waiting-list groups and 90.0% of the control group, while men represented 41.7% and 10.0%, respectively. The marked sex imbalance in the control group was by chance and only reflects the outcome of randomization. The mean BMI was high in all three groups, with mean values of 30.35 kg/m^2^ in the intervention and waiting-list groups and 30.99 kg/m^2^ in the control group, indicating obesity. Overall, groups showed very similar sociodemographic, nutritional, and anthropometric profiles.

Despite the initial amount of 22 older participants and depending on the timepoint, that number fluctuated. Thus, a maximum of twenty-two older people provided data at baseline, 21 at the time of intermediate assessment, and 19 and 20 at endline and follow-up timepoints, respectively.

### 4.3. Subjective Well-Being, Self-Esteem and Perceived Health

In line with the wait-list design, psychosocial outcomes were analysed by comparing participants exposed to the IGD (intervention and waiting-list groups) with control participants across baseline, intermediate, endline, and follow-up assessments. The global mixed linear models estimated for subjective well-being (WHO-5), self-esteem (Rosenberg scale), and perceived general health (EQ-5D) showed a convergent pattern (consistent positive directionality) of temporal change, with no evidence of differential effects associated with the intergenerational intervention (Table 2). In all three models, the main effect of time (pre vs. post), estimated for the control group, was positive and statistically significant in the case of subjective well-being (β = 1.537, *p* < 0.05) and self-esteem (β = 1.521, *p* < 0.01), indicating substantial increases in both dimensions over the period, regardless of membership in the intervention or waiting-list group. In contrast, in perceived general health (EQ-5D), the temporal effect, although positive, did not reach statistical significance, suggesting a more moderate and less robust improvement in this dimension.

The main group effect (intervention/waiting-list vs. control), interpreted as a difference in level at baseline, was not significant in any of the three models, indicating that both groups started from comparable levels after adjusting for age and degree of cognitive impairment (GDS score). Consistent with this result, the Time × Group interaction was not statistically significant for any of the variables analysed, implying that the pre-post trajectories of change were parallel in the intervention/waiting-list and control groups and that participation in the intergenerational dining room was not associated with a specific change in the temporal slope of the well-being and health indicators considered.

Finally, the covariates age and GDS score, included as time-invariant adjustments and centred on the sample mean, showed no significant associations with the results, reinforcing the preliminary interpretation that the variations observed were primarily due to a general temporal effect shared by both groups and not to structural differences attributable to the age or cognitive status of the participating older adults.

That said, a comparison of the estimated marginal means of subjective well-being, self-esteem, and perceived health between the control (CTR) and the intervention/waiting-list (INT) groups always yielded a favourable result for the latter, although only in the case of self-esteem did the difference reach statistical significance (CTR-INT = −1.415; *p* = 0.05). In general terms, this could be described as a favourable but non-statistically significant trend—a possible slight positive effect of the intergenerational dining room—which suggests that conducting another type of experiment with larger samples and longer follow-up periods would be valuable.

In contrast to the quantitative analysis, the qualitative analysis did show that participation in the IGD was associated with a perceived improvement in the emotional well-being, self-perception, and subjective health of older adults. Although the intensity of the effect varied depending on physical condition and personal context, most participants described a positive impact on their mood, daily motivation, and overall experience of well-being.

The elderly pointed out that the IGD was a space that reduced negative emotions, alleviated feelings of loneliness, and created an emotionally stimulating environment:

“I feel much better. Before, I was depressed, but now I feel more cheerful and supported”.

(OF#17)

“Here I feel better, supported and happier, with a purpose”.

(OF#25)

Participants also described improvements in their self-esteem, linked to the recognition they experienced during their interaction with the children and the active role they played during meals. This role seemed to reinforce their sense of usefulness and self-confidence:

“I feel happier, more independent”.

(OF#17)

Some older adults also linked their participation to positive changes in perceived health, including increased appetite, greater variety of foods eaten, and improved overall physical condition:

“I eat better now… vegetables, meat, fish. I’ve lost six kilos and I feel great”.

(OF#17)

Even those who were frail or tired reported that the experience was contributing to their emotional well-being, providing companionship and emotional support:

“I’m not always well, but I like coming here because I feel supported, and it shows”.

(OF#02)

The predominant emotions associated with intergenerational togetherness—joy, tenderness, and satisfaction—were identified as factors that directly influenced their daily well-being:

“I was happy to go, the children amuse me and make me feel good”.

(OF#25)

‘Just seeing their little faces makes me happy’.

(OF#24)

### 4.4. Food Intake

**Figure 2:**
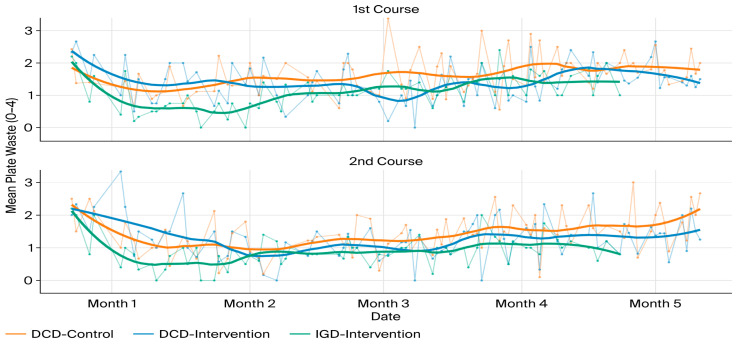
Time variation of plate leftovers (IGD vs. DCD). Note: Thick line: LOESS trend. Thin lines with dots: observed daily means. In this particular case, “Intervention” includes both intervention and waiting-list groups.

A total of 2946 photographs of first courses (1477) and second courses (1469) were taken before and after consumption, both at the IGD and in the day centre dining rooms (DCD) where only older adults were seated at the table. Figure 2 shows the changes in the amount of plate waste recorded over the study period.

Type III ANOVA tests revealed significant quadratic time components (F = 10.19; *p* < 0.005) indicating non-linear temporal trajectories. Therefore, mixed models incorporating quadratic components were applied to OF and LF data. Results highlighted significant and consistent effects for OFs associated with participation in the intergenerational mealtimes, compared to those demonstrated by LFs. The effect was particularly robust in the second course (β = −0.666; *p* < 0.001), indicating that, in the context of the pilot carried out, the differential impact of the intergenerational dining room on OFs, in terms of a reduction in leftover food in IGD versus DCD, was significantly greater than that shown by the children (IGD versus nursery school dining room) eating with them.

However, this article focuses specifically on what happened to older adults depending on the dining room used. In this regard, detailed analysis of the direct estimate using marginal measures made it possible to assess the effect of the IGD in the particular case of the OFs in the intervention and waiting-list groups. For the first course, the estimated marginal contrasts showed no statistically significant differences between the intergenerational dining room and the control dining room, either at the beginning (IGD–DCD = 0.18; *p* = 0.52) or at the end of the intervention (IGD–DCD = −0.14; *p* = 0.62). These results indicate that the potential effect of the intergenerational context on reducing plate waste in the first course was limited and not systematic among OFs. In the specific case of the second course, the estimated marginal contrasts of the mixed model made it possible to conclude that at the beginning of the intervention there were no significant differences between the intergenerational dining room and the control dining room (IGD-DCD = −0.31; *p* = 0.12); however, at the end of the pilot, a significant reduction in leftover food from the second course was observed in the intergenerational dining room (IGD–DCD = −0.52; *p* = 0.01), indicating possibilities for a progressive and sustained improvement in the food intake of OFs in the intergenerational context.

The same model was applied to mean plate waste—the average of the first and second course plate waste combined. The marginal contrasts estimated for OFs did not show significant differences between the intergenerational dining room and the control dining room either at the beginning of the intervention (IGD–DCD = −0.05; *p* = 0.81) or at the end of the intervention (IGD–DCD = −0.32; *p* = 0.12), although the latter contrast suggests a trend towards less leftover food in the intergenerational context. This pattern is consistent with the concentration of the intervention effect on the second course, whose signal is attenuated when averaging both components of intake.

Finally, upon entering the follow-up phase—right after leaving the intergenerational dining room—the contrasts of estimated marginal means revealed that the differences between the intervention/waiting-list groups and the control group in the mean residuals were not statistically significant (IGD-DCD = −0.14, *p* = 0.49). However, at the end of the follow-up period, OFs in the intervention/waiting-list groups showed significantly lower levels of plate waste than their counterparts in the control group (IGD-DCD = −0.57, *p* = 0.005). This pattern may suggest that the beneficial effect associated with participation in the intergenerational dining room—mainly due to second course consumption—not only persisted after leaving it, but tended to consolidate and amplify over time, in contrast to the stability observed in the control group.

Qualitative data analysis revealed that many older adults reported that, since participating in the IGD, they were eating better and with greater appetite, attributing this change to the emotional atmosphere and the company of the children. Some described specific improvements in the variety and volume of food consumed, especially those foods that were less commonly accepted:

“Yes, I eat better now… vegetables, meat, fish. I’ve lost six kilos and I feel great.”

(OF#17)

“The children bring me joy… and that makes me enjoy eating more.”

(OF#08)

Emotional well-being emerged as a key factor in improving nutrition. Many older adults indicated that eating in the IGD gave them encouragement, serenity, and motivation, conditions that had a positive impact on their willingness to eat:

“I used to be depressed, but now I feel more cheerful and supported.”

(OF#17)

“I feel better here, supported and happier, with a purpose.”

(OF#25)

Several participants explicitly linked their emotional improvement to a better relationship with food, noting that feeling better also led to eating better:

“I feel happier, more independent.”

(OF#17)

Interaction with children also acted as a driver for healthy eating habits. In their role as companions and mentors, many older adults reinforced appropriate consumption norms, which had a knock-on effect on their own behaviour:

‘I told them they shouldn’t waste food… and I ate better myself.’

(OF#25)

‘Seeing them eat with gusto, I also ate more.’

(OF#24)

The emotional component of intergenerational experiences—joy, tenderness, affection, and recognition—was equally decisive. Meals and mealtime became a rewarding relational space, which stimulated appetite and encouraged them to eat everything on the plate:

“I was happy to go; the children amuse me and make me feel good.”

(OF#25)

“Just seeing their little faces makes me happy.”

(OF#24)

Even among those who reported physical limitations or fatigue, the experience had a positive effect, promoting a desire to participate and pay more careful attention to their own eating, despite their vulnerability:

‘I’m not always well, but I like coming here because I feel supported, and it shows.’

(OF#02)

## 5. Discussion

Since this is an external pilot and feasibility study aiming to guide a definitive randomised controlled trial (RCT) [32], a note of caution is warranted in interpreting intervention effects. The study was not powered to establish efficacy, nor to provide definitive estimates of effectiveness under routine conditions. Rather, its purpose was to explore feasibility parameters and to obtain preliminary estimates of the direction and magnitude of potential effects. Therefore, both significant and non-significant findings should be understood as inconclusive results to avoid overinterpretation.

The increases observed in subjective well-being and self-esteem, in the intervention, waiting-list, and control groups, reflect a general trend that has been detected in interventions promoting meaningful social connections in old age. Previous studies show that relational care, mutual support, and reciprocity contribute directly to well-being in older adults [14,15]. Clearly, the IGD reproduces these conditions: older adults reported feeling accompanied, valued, and emotionally stimulated, which is in line with results obtained in other intergenerational meal programmes [13], as well as an indicator of improved quality of life [33].

Although quantitative analyses did not reveal a differential effect of the intergenerational dining room on the slope of change, older adults in the intervention and waiting-list groups were observed to maintain consistently higher levels of well-being, self-esteem, and perceived general health. The absence of statistically significant Time × Group interactions in the psychosocial outcomes should be interpreted with caution in light of the pilot and feasibility nature of the study. In this context, the mixed-effects models were primarily intended to explore directionality rather than to provide definitive estimates of efficacy. It is therefore plausible that subtle intervention-related changes in subjective well-being, self-esteem, and perceived health may not have been fully captured within the time frame and sample size available.

Qualitative evidence helps explain this discrepancy: participants described the dining room as a space that ‘livens them up,’ provides a sense of purpose, and reduces loneliness, factors that may not be adequately captured statistically in the repeated measurements during this short pilot. But they do reflect relational mechanisms very pertinent to subjective health, which underlines that intergenerational dining may serve as an environment for positive mental and emotional health, consistent with recent research linking shared meals with improvements in mental well-being, social connection, and mood [34].

One of the most encouraging findings was the significant reduction in leftover food among older adults, specifically in the second course and showing a promising trend in the average first and second course plate waste. This effect suggests preliminary indications (rather than evidence of sustained causal impact) of a behavioural change, visible both during the intervention and in the follow-up phase.

The literature indicates that eating in the company of others increases enjoyment, attention to food, and adherence to healthy guidelines [34,35]. This study’s qualitative results strengthen this interpretation: many older adults directly linked the company of children with increased appetite, a larger variety of foods eaten, and greater willingness to finish the second course.

It is interesting to note as well that a relevant mechanism emerged: the ‘mirror effect’ or reciprocal modelling. While playing an educational role towards children, older adults also reinforced their own eating habits, a phenomenon observed in other intergenerational interventions [36].

Taken together, these results show that the emotional and relational dimension of food—a central element of the intergenerational dining model—might act as a catalyst for practical and measurable dietary changes. In a field with little experimental research, this evidence is particularly valuable.

When the quantitative and qualitative results are integrated, it becomes clear that the impact of the intergenerational dining room might be explained by a series of mechanisms frequently described in the intergenerational literature. For instance, some older interviewees acknowledge increase in appetite, a finding that is aligned with observed reduction in second-course leftover food. These mechanisms include reciprocity, the exercise of meaningful roles, mutual recognition, shared learning, and the construction of stable emotional bonds [11,37]. In this study, these processes seem to manifest themselves particularly clearly in two fundamental areas: on the one hand, in emotional well-being, as daily interaction with children offered older adults a source of vitality, companionship, and a sense of purpose that is reflected in improved mood and daily motivation; and on the other hand, in eating behaviour, where the symbolic and emotional dimension of shared meals promoted a more positive relationship with food and contributed to reducing food waste. This combination of factors coincides with theoretical approaches that view commensality as a relational practice influential in nutrition, identity, and care. As Fischler points out, “commensality produces bonding” [38] (p. 533).

The findings of this pilot study provide novel and promising evidence on the potential of intergenerational dining rooms as health promotion environments in day centres. In contexts where loneliness, low food intake, and lack of motivation to eat are common challenges [39], IGD may improve both the emotional well-being of older adults and their relationship with food.

### 5.1. Strengths and Limitations

This study has several notable strengths. First, it employs a wait-list randomised controlled design, something still quite uncommon in research on intergenerational programmes, particularly intergenerational commensality. Second, the use of a sequential explanatory mixed-methods approach permits an integrated understanding of both measurable outcomes and the relational and emotional mechanisms underlying observed changes. The convergence between quantitative trends and qualitative accounts enhances the interpretability of the findings.

A further strength lies in the inclusion of objective, behaviour-based measures of food intake, namely photographic plate-leftover assessment using the Comstock method, which showed excellent inter-rater reliability. Compared with self-reported dietary data, this approach provides a more robust and ecologically valid indicator of actual eating behaviour. In addition, the intervention was embedded in the routine operation of a real-world intergenerational centre, thereby enhancing ecological validity and practical relevance.

Finally, the post-intervention follow-up phase allowed for an initial assessment of whether changes in food consumption persisted beyond active exposure to the intergenerational setting.

Several limitations must also be acknowledged. The small sample size, inherent to pilot and feasibility studies, limited statistical power to detect small or moderate differential effects between groups, particularly for psychosocial outcomes such as well-being and self-esteem. However, CONSORT 2010 explains that pilot trials are usually underpowered to do formal hypothesis testing [18]. Similarly, the relatively short duration of the intervention may have constrained the detection of longer-term or cumulative effects. The reliance on self-reported measures for subjective well-being, self-esteem, and perceived health may have introduced response bias and may not fully capture subtle or incremental changes in relational well-being.

Moreover, the study was conducted in a single intergenerational centre, and therefore its generalizability to other organizational, cultural, or institutional contexts is very limited. Also, participation was voluntary, so a selection bias toward more motivated or socially engaged individuals cannot be ruled out. These limitations, however, are consistent with the exploratory aims of a pilot and feasibility study and primarily serve to inform the design, measurement strategy and sample size estimation of future large-scale trials.

### 5.2. Implications and Future Directions

The findings of this pilot and feasibility study have several implications for research, practice, and policy. From a research perspective, the results support the conceptualization of intergenerational commensality as a promising relational and emotional intervention rather than solely a nutritional one. However, the absence of strong differential effects on self-reported well-being, combined with changes in food consumption and rich qualitative evidence, suggests the need for outcome measures more sensitive to relational processes, such as sense of purpose, social connectedness, and affective engagement.

From a practical standpoint, intergenerational dining models appear to represent a feasible and low-threshold strategy for enhancing eating experiences and reducing food waste in day care settings for older adults, particularly where intergenerational co-location already exists. The observed reductions in plate leftovers also point to potential benefits aligned with sustainability goals, linking older adult care, nutritional health, and responsible food consumption. In terms of operational considerations when considering scalability to other centres, this feasibility study has produced some guidelines (not the object of this paper) highlighting attention to practical constraints such as space limitations, sample attrition because of older adult fatigue, and compatibility of older people and children lunch schedules.

Future research should aim to scale up this model through multicentre studies, ideally using cluster-randomised designs to account for institutional effects. Longer follow-up periods are needed to assess the durability of behavioural changes and their potential impact on nutritional status and health trajectories. Incorporating biomedical and functional indicators (e.g., nutritional biomarkers, physical functioning) into the models would further strengthen causal inferences. Overall, aside from the question of whether intergenerational commensality works, future research should seek to understand how, for whom, and under which institutional conditions it produces sustainable benefits for health, well-being, and care quality in later life.

## 6. Conclusions

This pilot randomised wait-list trial provides preliminary evidence regarding the feasibility and potential effects of implementing a model of intergenerational commensality in adult day care settings. While quantitative analyses did not demonstrate statistically significant differential effects on psychosocial indicators among OFs attending the IGD, qualitative findings suggest perceived improvements in older people’s emotional well-being and relational engagement.

The study also indicates a potential reduction in plate waste during IGD exposure periods. Given the exploratory nature and limited sample size, findings should be interpreted cautiously. Adequately powered trials with larger and longer duration are required to confirm these preliminary observations and thus determine whether sustained participation in an intergenerational dining room produces measurable psychosocial and eating habit effects.

Overall, this pilot study supports the idea that intergenerational dining rooms are a promising intervention for promoting well-being, self-esteem, and better eating among older people, offering a relational approach that might be helpful in addressing key policy challenges stemming from the contemporary ageing process.

## Figures and Tables

**Table 1:** Descriptive characteristics of the OF sample (*n* = 22).

Variable	Intervention and Waiting-List Groups (Groups)		Control Group		Baseline Comparison		
					Test	Estimate	*p*-Value
Age, mean years (sd)	82.83 (5.01)		82.80 (4.69)		Welch *t*-test	0.007	0.987
Sex, *n* (%) (female)	7 (58.3)		9 (90.0)		Fisher exact	5.924	0.162
Sex, *n* (%) (male)	5 (41.7)		1 (10.0)		Mann–Whitney U	−0.250	0.334
Length of time in day care (months)	19.58 (25.53)		22.80 (15.59)		Welch *t*-test	−0.139	0.742
MNA-SF	11.50 (2.02)		11.80 (2.15)		Welch *t*-test	0.074	0.853
GDS	2.96 (1.63)		2.85 (1.06)		Mann–Whitney U	0.033	0.893
EAT-10	1.25 (2.53)		1.20 (2.57)		Welch *t*-test	0.337	0.405
Height (m)	1.57 (0.11)		1.54 (0.07)		Welch *t*-test	0.038	0.929
Weight (kg)	74.25 (18.01)		73.53 (18.26)		Welch *t*-test	−0.096	0.822
BMI (kg/m^2^)	30.35 (6.19)		30.99 (6.60)		Welch *t*-test	−0.230	0.607
Calf circumference (cm)	35.92 (3.99)		37.35 (7.79)		Welch *t*-test	0.007	0.987
	Baseline	Endline	Baseline	Endline			
Subjective well-being (WHO-5)	8.50 (2.51)	12.42 (2.39)	6.89 (1.83)	10.00 (3.20)	Welch *t*-test	0.703	0.159
Self-esteem (Rosenberg scale)	14.12 (2.03)	16.75 (0.87)	13.00 (1.41)	16.00 (1.41)	Welch *t*-test	0.617	0.214
Perceived general health (EQ-5D)	6.06 (2.18)	7.42 (1.40)	5.67 (1.32)	6.50 (1.27)	Welch *t*-test	0.212	0.664

Note: Values are expressed as mean (SD) unless otherwise stated. Baseline and endline values correspond to measurements at time 0 and time 2, respectively. BMI = Body Mass Index; MNA-SF = Mini Nutritional Assessment–Short Form; GDS = Global Deterioration Scale; EAT-10 = Eating Assessment Tool.

**Table 2:** Fixed effects from global lineal mixed-effects models.

	Subjective Well-Being (WHO-5)			Self-Esteem (Rosenberg Scale)			Perceived General Health (EQ-5D)		
Effect	β	SE	t	β	SE	t	β	SE	t
Intercept	7.270 ***	0.691	10.523	12.888 ***	0.431	29.906	5.705 ***	0.375	15.220
Time (pre vs. post), control group	1.537 *	0.642	2.395	1.521 **	0.554	2.748	0.460	0.260	1.769
Group (intervention/waiting-list vs. control)	1.609	1.038	1.550	1.328	0.696	1.909	0.232	0.540	0.429
Age (centred)	−0.091	0.095	−0.961	−0.016	0.054	−0.289	−0.002	0.053	−0.044
GDS (centred)	0.146	0.339	0.431	0.171	0.201	0.853	0.252	0.187	1.345
Time x Group interaction	0.079	1.059	0.074	0.087	0.910	0.096	0.383	0.430	0.890

Note. B = Unstandardized coefficient; SE = Standard error. Time is coded as pre (0) and post (1). Group is coded as control (0) and intervention/waiting-list (1). Age and GDS were centred at the sample mean. Models include random intercepts for participants. * *p* < 0.05, ** *p* < 0.01, *** *p* < 0.001.

## Data Availability

The data presented in this study are available on request from the corresponding author. The data are not publicly available due to privacy and ethical restrictions.

## Supplementary Materials

The following supporting information can be downloaded at: https://www.mdpi.com/article/10.3390/healthcare14050635/s1, Semi-structured script for OF interviews.
