# Supplementary material for: Intergenerational Mealtimes in Adult Day Care Settings: Impact of a Pilot Randomised Control Study on the Well-Being, Health, and Food Intake of Older Adults
Source: Healthcare (Basel). 2026 Mar 3;14(5):635. doi: 10.3390/healthcare14050635 (PMC12984263; doi:10.3390/healthcare14050635)
Supplement: Supplementary file 1 [file healthcare-14-00635-s001.zip › healthcare-4102886-supplementary.pdf]

Semi-structured script for OF interviews

Initial contextualization

I would like us to talk about your experience so far in the Intergenerational Dining Room (IGD). You've been eating at the IGD for a few weeks now. I'd like to know how things are going for you so far. I'd also like you to tell me about any differences you may be noticing between eating with the "Little Friends" (LFs) and eating with your older peers, as you'd been doing before getting involved in the IGD.

Introductory questions

- In general, how would you describe your experience of eating with the LFs?
- What do you like most about participating in the IGD?
- What aspects do you find most difficult or least enjoyable?
- Do you think there are differences between eating with the LFs and eating with your older peers at the Day Center's dining room? If so, how is eating in one place different from eating in the other?
- How do you view the other "Older Friends" who are with you in the IGD?
- Would you say that you prefer eating with the LFs or with other older people?
- Would you like to keep eating at the IGD?

Specific questions

Relationships, care, and eating

- How is your daily relationship with the LFs like during meals?
- Did you feel that they cared for you or that you cared for them at any time?
- Has this experience of eating at the IDG changed the way you relate to other older people? To other children?
- Have you talked about this experience with people around you? Who? What did you tell them?

Eating habits and diet

- Did you learn anything new about food, health, or eating habits during your participation?
- Is there anything about your diet that you have changed or that you see differently thanks to this experience?

Physical/emotional health and well-being

- Has participating in the IGD changed your health in any way?
- How did you feel physically and emotionally before, during, and after meals with the LFs? For example, have you felt more tired eating with the children?
- What about your overall sense of well-being?

Intergenerational views

- How did you view the LFs at the start of the project and how do you view them now?
- Has your way of thinking about children or other generations changed because of your participation in the IGD?

In closing

- What have you taken away from this experience so far?
- Is there anything you think we could do to improve the project?
- Do you feel like continuing in the project during the remaining weeks?
